# Deficiency of the Alzheimer's disease gene SORL1 impairs lysosomal enzyme trafficking in human microglia

**DOI:** 10.1002/alz70855_099991

**Published:** 2025-12-23

**Authors:** Jessica E. Young, Suman Jayadev, Sonia B Sidhu, Swati Mishra, Nader Morshed, Beth Stevens

**Affiliations:** ^1^ University of Washington, Seattle, WA, USA; ^2^ University of Washington, SEATTLE, WA, USA; ^3^ Broad Institute of MIT and Harvard, Cambridge, MA, USA

## Abstract

**Background:**

The *SORL1* gene encodes the sortilin related receptor protein SORLA, a sorting receptor that regulates endo‐lysosomal trafficking of various substrates. Loss of function variants in SORL1 are causative for Alzheimer's disease (AD) and decreased expression of SORLA has been repeatedly observed in human AD brains. SORL1 is highly expressed in the central nervous system, including in microglia, the tissue resident immune cells of the brain. Loss of SORLA leads to enlarged lysosomes in hiPSC‐derived microglia like cells (hMGLs). However, how SORLA deficiency contributes to lysosomal dysfunction in microglia and how this contributes to AD pathogenesis is not known.

**Method:**

hiPSCs with loss of *SORL1* were generated using CRISPR/Cas9 technology and differentiated into microglia like cells (hMGLs). Lysosomal degradation, enzyme activity and intracellular trafficking of the lysosomal enzymes HEXB, Cathepsin B and Cathepsin D, lysosomal exocytosis and lysosomal accumulation of Aß (1‐42) and synaptosomes were analyzed in *SORL1* KO hMGLs relative to their isogenic control hMGLs. Additionally, phagocytosis of Aß (1‐42) and synaptosomes and inflammatory response were evaluated.

**Result:**

In this study, we show that loss of SORLA results in decreased lysosomal degradation and lysosomal enzyme activity due to altered trafficking of lysosomal enzymes in hMGLs. Phagocytic uptake of fibrillar amyloid beta 1‐42 and synaptosomes is increased in SORLA deficient hMGLs, but due to reduced lysosomal degradation, these substrates aberrantly accumulate in lysosomes. An alternative mechanism of lysosome clearance, lysosomal exocytosis, is also impaired in SORL1 deficient microglia, which may contribute to an altered immune response. Overall, these data suggest that SORLA has an important role in proper trafficking of lysosomal hydrolases in hMGLs, which is critical for microglial function.

**Conclusion:**

Our findings further substantiate the microglial endo‐lysosomal network as a potential novel pathway through which SORL1 may increase AD risk and contribute to development of AD. Additionally, our findings may inform development of novel lysosome and microglia associated drug targets for AD.

